# Emergence of Cerebral Mucormycosis in the Post-COVID Period: A Detailed Analysis of Risk Factors, Clinical Progression, and Management of This Opportunistic Fungal Infection

**DOI:** 10.7759/cureus.31220

**Published:** 2022-11-07

**Authors:** Masum Patel, Jigar Panchal, Chetna Desai, Jaimin Shah, Bela Prajapati, Shubham Patel

**Affiliations:** 1 Medicine, B.J. (Byramjee Jeejeebhoy) Medical College, New Civil Hospital Asarwa, Ahmedabad, IND; 2 Pharmacology, B.J. (Byramjee Jeejeebhoy) Medical College, New Civil Hospital Asarwa, Ahmedabad, IND; 3 Neurosurgery, B.J. (Byramjee Jeejeebhoy) Medical College, New Civil Hospital Asarwa, Ahmedabad, IND; 4 ENT, B.J. (Byramjee Jeejeebhoy) Medical College, New Civil Hospital Asarwa, Ahmedabad, IND

**Keywords:** cavernous venous sinus thrombosis (cvst), carotid artery thrombosis, angioinvasive mucormycosis, post covid-19 rhino-orbito-cerebral mucormycosis (rocm), paranasal sinus diseases, covid-19 india, opportunistic mycoses, amphotericin, rhinocerebral mucormycosis

## Abstract

Background: An epidemic of Mucorales was reported following the second wave of COVID-19 in India, and intracranial extension of the same was one of the most dreadful complications.

Methods: A total of 62 patients with cerebral mucormycosis were recruited and followed up till 12 weeks to evaluate the risk factors, incidence, clinical manifestations, management, and prognosis of cerebral mucormycosis.

Findings: A median age of 51.5 years with male predominance (74%) was noted. The majority of subjects reported a history of COVID infection (93.5%) and diabetes mellitus (83.87%). The first symptom of mucormycosis appeared after a mean period of 17.63 ± 8.9 days following COVID. Facial swelling and ptosis were the most common symptoms. Only 55% of patients had neurological presentations, and hemiparesis was the most common neurological sign (30.6%). Radiologically, the involvement of maxillary sinus (90.32%) and ethmoid sinus (87.10%) was commonly noted. Cerebral findings included temporal lobe (50%) and parietal lobe (30.06%) involvement, cavernous sinus thrombosis (30.06%), and internal carotid artery thrombosis (22.58%). Acute cerebral infarction was notable in 37% of subjects (p-value=0.0015, significant association with the outcome). Conventional and liposomal amphotericin B were used in 91.94% and 53.23% of patients, respectively. Retrobulbar amphotericin injections used in 11.3% of subjects significantly affected the outcome (p-value=0.03, significant). Posaconazole step-down therapy was used in 72.5% of subjects (p-value=0.0005, significant). Surgical interventions were performed in 53 (85.48%) subjects (p-value=0.004, significant). Functional endoscopic sinus surgery was the most common (in 64.52% of subjects), followed by maxillectomy (20.97% of subjects) and craniotomy (17.7% of subjects). At the end of 12 weeks, 33.87% of patients died and 59.68% were alive; the rest (6.45%) were lost to follow-up.

Interpretation: The absence or late presentation of neurological symptoms led to a delayed diagnosis of cerebral mucormycosis. The presence of acute cerebral infarction indicated a worse prognosis. However, there was a significant influence of step-down posaconazole therapy, retrobulbar amphotericin injections, and surgical intervention on the prognosis of cerebral mucormycosis.

## Introduction

Background and rationale

Recently, a potentially fatal invasive secondary fungal infection has emerged in the form of COVID-19-associated mucormycosis (CAM), due to opportunistic infection by *Rhizopus* or *Mucromycetes*. Prior incidence of mucormycosis was only about 50 cases per year, as reported in an 18-month study by A Chakrabarty et al. [1]. But the incidence of CAM was significantly higher during the second wave of COVID-19, with about 14,872 cases as of May 28, 2021, in India. The highest number of cases of mucormycosis in patients with active and recovered COVID-19 (n=3726) were reported in the state of Gujarat [2]. COVID-19 patients are more susceptible to opportunistic fungal infections due to the immune dysregulation caused by iatrogenic immunosuppression (via corticosteroids or undefined antibiotic treatment), uncontrolled diabetes mellitus, use of invasive or noninvasive ventilation, and other pre-existing conditions [3,4].

The fungus initially invades the nasal cavity and paranasal sinuses with presenting features similar to acute sinusitis but can often lead to angioinvasion and subsequent thrombosis. It rapidly spreads to orbital and cerebral sites, leading to deteriorating clinical outcomes [5,6]. Direct cerebral involvement is rare but possible if the infection spreads posteriorly from the orbit or sinuses to the central nervous system. The angioinvasive nature of the fungus may result in thrombosis of the cavernous sinus and internal carotid artery as well [7,8]. First-line treatment with high-dose liposomal amphotericin B is strongly recommended, while a step-down therapy of isavuconazole and posaconazole is suggested with moderate strength [9]. Rinsing surgical areas topically with amphotericin B solution has shown clinical improvements without resulting in any of its major side effects [10-13].

Early diagnosis of cerebrovascular mucormycosis remains difficult due to a lack of specific neurological symptoms early in the course of the disease. A combination of craniotomy and resection of the brain abscess or a pathological autopsy can confirm the disease. Despite surgical intervention and antifungal medications, the mortality of patients with cerebral mucormycosis is recorded to be fairly high (especially if the disease progressed to later stages) [7]. 

Objectives

Cerebral mucormycosis is a rare disease; therefore, the alarming rise in the incidence of CAM following the COVID-19 pandemic is intriguing. Hence, our study objectives were as follows:

1. To study the risk factors for the occurrence of mucormycosis and its cerebral extension.

2. To record the temporal presentation of signs and symptoms of the subjects.

3. To evaluate the impact of various interventions (including antifungal agents and procedures) on the clinical outcome of the disease.

## Materials and methods

Study design

It is a mixed (retrospective and prospective) type of cohort study, centered in the mucormycosis ward in Civil Hospital Ahmedabad, India. Cases of cerebral mucormycosis were enrolled as per the following selection criteria mentioned below and were followed up for 12 weeks (from 28th June to 20th September 2021). Sampling size was dictated by the number of cerebral mucormycosis patients meeting the inclusion criteria in the hospital during the study period.

Inclusion criteria

· Patients of age 18 years and above of either gender.

·Confirmed diagnosis of mucormycosis by mandatory presence of clinical manifestations and radiological findings (either computed tomography [CT] scan or magnetic resonance imaging [MRI]); and fungal evidence on either culture or histopathological examination of the biopsy samples.

· Confirmed diagnosis of cerebral involvement by any one of the two:

1. CT scan - irregular low-density areas inside the cerebral parenchyma.

2. MRI - low T1-weighted imaging signals and high T2-weighted imaging signals.

Study method

After acquiring permission from "The Institutional Ethics Committee B. J. Medical College & Civil Hospital, Ahmedabad" (Ref. No. EC/Approval/71/2021/12/06/2021), subjects were enrolled according to the selection criteria for cases and controls. The investigator elucidated the procedures and objectives of the study and obtained informed written consent from each subject in the vernacular language.

Baseline data like demographic details and medical history were noted. COVID-related history included RT-PCR (real-time reverse transcription polymerase chain reaction) confirmation, HRCT (high-resolution computed tomography) scores, and medical interventions (all in accordance with revised guidelines on clinical management of COVID-19 [14]). In subjects with COVID-positive tests, the temporal relation between COVID, mucormycosis, and its cerebral extension was noted in terms of time spans between each. While recruiting the subjects, the diagnosis of mucormycosis and its cerebral extension was confirmed, and signs and symptoms, microbiological examination, blood investigations, and radiological examinations were recorded on the first visit. Primary antifungal therapy included conventional amphotericin B (cAmB) (1-pint nasal saline followed by 50 mg intravenous amphotericin slowly over 4 hours for a target duration of 14 days) and/or liposomal amphotericin (250 mg intravenous in 5% dextrose solution slowly over 2-3 hours for a target duration of 21 days). After hospital discharge, the patients were prescribed step-down posaconazole (three tablets daily, 100 mg each), and it was continued till the absence of fungal evidence in biopsy samples. A few subjects were given retrobulbar amphotericin injections (10 mg dissolved in 2 ml solvent each day for four days). All the patients were assessed to note clinical progression, surgical and pharmacological management, and any adverse events. After discharge, step-down posaconazole therapy was prescribed and patients were followed up every two weeks. The total follow-up period was 12 weeks, and the final outcome (alive/dead) was recorded.

The final data of all the subjects were recorded in Microsoft Excel 2019. The quantitative variables were assessed with the help of an unpaired T-test if normally distributed and the Mann Whitney U test if not normally distributed. Categorical variables were assessed with the help of a chi-square test. An association was considered significant if the p-value was less than 0.05. The final data were compiled and expressed in the forms of frequency/proportion/mean or median.

## Results

Demographic details

A total of 62 patients with cerebral extension of mucormycosis were enrolled in the study. The majority of subjects were males (n=46,74.2%). The age of the subjects ranged from 20 to 84 years (median 51.5 years, mean 50.82±11.95). The most common age group affected was 45-64 years (n=35, 56.5%). Tobacco chewing was found to be the most common substance use disorder (n=27 subjects, 43.6%) followed by tobacco smoking (n=10 subjects, 16.13%), and alcohol consumption (n=6 subjects, 9.7%). The body mass index (BMI) of the subjects ranged from 15.1 to 34.4 (median 23.8). The majority of subjects (n=36, 58.06%) recorded normal BMI. Other plausible risk factors are enlisted in Table 1.

**Table 1:** Plausible risk factors in 62 patients of cerebral mucormycosis BMI, body mass index; DM, diabetes mellitus.

Comorbidities	Number of patients (%) (n=62)
Occupational exposure to soil	23 (37.1%)
BMI	
Overweight	14 (22.58%)
Obese	8 (12.9%)
DM (prior to COVID)	26 (41·94%)
DM (post COVID)	52 (83.87%)
Hypertension	13 (20·97%)
Diabetic ketoacidosis	02 (3.23%)
Tuberculosis	01 (1.61%)

COVID-19-related history

All 62 subjects reported being symptomatic with flu-like illness in the past two months before the diagnosis of mucormycosis. But only 58 (93.55%) out of the 62 subjects were positive for COVID in RT-PCR. The severity of the COVID infection was assessed by duration of COVID-19 infection, HRCT score, hospitalization requirement (other subjects were treated ambulatory), and oxygen therapy. Oxygen therapy was required in 19 (30.65%) for an average period of 8.63±13.44 days. Treatment for COVID-19 was taken by 54 (87.10%) patients. Details of COVID history are presented in Table 2.

**Table 2:** COVID-related history (n=58 subjects out of total 62 subjects) *A semi-quantitative CT score was calculated based on the extent of lobar involvement (0: 0%; 1: < 5%; 2: 5-25%; 3: 26-50%; 4: 51-75%; 5: >75%; range 0-5; global score 0-25) [15]. **Included either oral/intravenous forms of dexamethasone or methyl prednisone. HRCT, high-resolution computed tomography.

COVID-related parameters	Values
Mean duration (n=58)	13.96±5.02 days
Median	12 days
HRCT scores* (n=58)	
<8 (mild)	19
8-17 (moderate)	34
>17 (severe)	5
Hospitalization due to COVID	24 (38.71%)
Oxygen therapy	19 (30.64%)
Drug treatment received	54 (87.10%)
Antiviral drugs	39 (62.90%)
Antibacterial drugs	43 (69.35%)
Zinc supplements	27 (43·55%)
Iron supplements	05 (8.06%)
Steroids**	38 (61·29%)
Duration of steroid therapy	6.3 ± 2.7 days
Median	5 days
History of COVID vaccine	
First dose	14 (22.58%)
Second dose	01 (1.61%)

The chronological appearance of signs and symptoms

**Figure 1:**
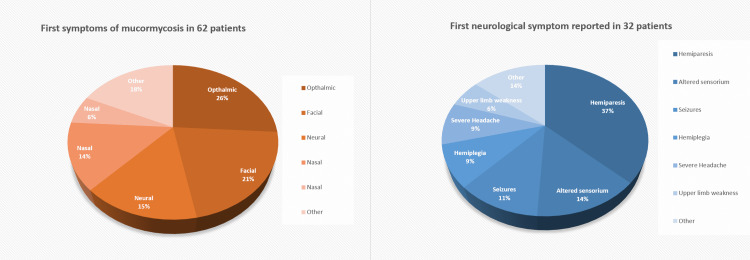
First plausible mucormycosis symptoms (n=62) and the first neurological symptoms (only in n=34, 54.8%) reported in 62 subjects Ophthalmic symptoms - periorbital swelling, orbital pain, chemosis; facial symptoms - facial swelling or pain; oral symptoms - dental pain, painful gums, gingival swelling, throat pain; neural symptoms - ptosis, hemiplegia; nasal symptoms - bleeding, pain, blockage; others severe headache or fever.

The median time lag between COVID infection (considered RT-PCR-positive test date) and the appearance of the first symptom of mucormycosis was found to be 16 days. Eighteen (29.03%) patients were symptomatic for mucormycosis even before the resolution of COVID infection (considered RT-PCR-negative test date). Only 34 (54.84%) patients presented with neurological symptoms throughout the course of their cerebral extension of Mucorales. The mean "time lag" between the first symptom of mucormycosis and the first neurological symptom was 19 days (median). We noted the first plausible mucormycosis symptom (n=62) and the first neurological symptom (only in n=34, 54.8%) reported by the patient. A graphical representation of the same is provided in Figure 1.

Clinical features

**Figure 2:**
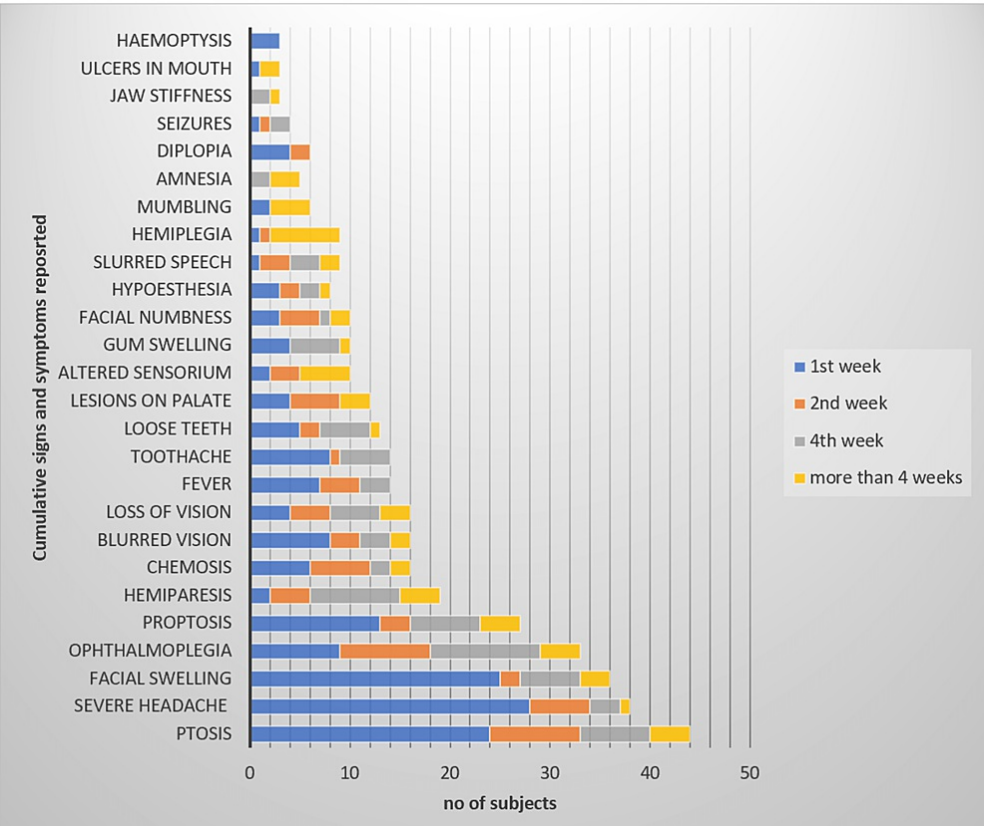
Progression of signs and symptoms of the disease

We recorded the presentation of clinical signs and symptoms in terms of both the frequency and the time of appearance; among them, ptosis was the most common sign (n=44, 70.97%) while severe headache (n=38, 61.29%) was the most common presenting symptom. Out of 62 patients, only 34 (54.84%) patients presented with neurological signs and symptoms. Hemiparesis was the most common neurological sign in 19 (30.65%), followed by altered sensorium in 12 (19.35%). Figure 2 provides a detailed record of systemic and neurological signs and symptoms along with their appearance.

Radiological findings

Radiologically, the maxillary sinus was the most affected being involved in 56 (90.32%) patients followed by the ethmoid sinus in 54 (87.10%). The orbital invasion was observed in 44 (70.97%) patients and the optic nerve was affected in 25 (40.32%) patients. In the brain, the temporal lobe was involved in 31 (50%) patients followed by the parietal lobe in 19 (30.06%) patients and the frontal lobe in 15 (24.19%) patients. Internal carotid artery thrombosis was reported in 14 (22.58%) patients and cavernous sinus thrombosis was observed in 19 (30.06%) patients. Other findings of MRI and CT scans are presented in Table 3.

**Table 3:** Anatomical invasion of mucormycosis as per diagnostic modality (n=62) *Other lobes include parietooccipital, temporoparietal, front parietooccipital, and front parietotemporal. ICA, internal carotid artery; EOM, extraocular muscle.

Anatomical location	Involved in	Unilateral	Bilateral
Paranasal sinus			
Maxillary	56 (90·32%)	25	31
Ethmoid	54 (87.10%)	24	30
Sphenoid	53 (85·48%)	17	36
Frontal	34 (54·84%)	19	15
Orbital invasion	44 (70·97%)	41	04
Optic nerve involvement	25 (40·32%)	24	01
Trigeminal nerve involvement	6 (9.68%)	06	00
Masticator thickening	13 (20·97%)		
EOM thickening	3 (4·84%)		
Mastoiditis	4 (6.45%)		
Brain involvement: Temporal lobe	31 (50%)	29	02
Parietal lobe	19 (30.06%)	14	05
Frontal lobe	15 (24.19%)	12	03
Basi frontal lobe	12 (19.35%)	09	03
Occipital lobe	11 (17.74%)	10	01
Frontoparietal lobe	10 (16.13%)	08	02
Other lobes*	08 (12.90%)	07	01
Cavernous sinus thrombosis	19 (30%)	17	02
ICA thrombosis	14 (22.58%)	11	03
Acute infarcts	24 (38.71%)		
Pachymeningitis	13 (20.97%)		
Cerebral abscess	13 (20.97%)		
Meningitis	12 (20.97%)		
Bone erosion	11 (17.74%)		

Pharmacotherapy

The study setting was in a tertiary care hospital and hence prompt antifungal therapy was started in all patients (n=62, 100%). cAmB was administered in 57 (91·94%) of the 62 patients. The mean duration of conventional amphotericin therapy was noted to be 14·65±8.81 days. 

Liposomal amphotericin was given to a total of 33 (53.23%) subjects, either as primary antifungal or after completing prescribing cAmB. The extent of cerebral extension and the potential risk of renal toxicity were primary factors in deciding the right antifungal drug. Amphotericin B-lipid complex (ABLC) (n=5,8.06% subjects), itraconazole (n=2 subjects), and voriconazole (n=2 subjects) were also used intermittently. Retrobulbar amphotericin B injections were used in 8 (12.9%) patients. Forty-seven (75.8%) subjects were prescribed posaconazole after completing the prescribed dose of amphotericin. All the patients were followed up every 15 days after discharge, and posaconazole therapy was continued till the absence of fungal evidence in biopsy samples. Overall, posaconazole (p-value=0.0005) and retrobulbar amphotericin administration (p-value=0.03) had a statistically significant effect on the clinical outcome. Other subjects were given alternate forms of amphotericin like liposomal amphotericin/ABLC. Other drugs like antibacterial agents, antiplatelet drugs, anticoagulants, antacids, antiemetics, analgesics, and sedatives were prescribed alongside the primary treatment as and when necessary.

Laboratory abnormalities after treatment initiation with conventional amphotericin: We closely observed the adverse effects (AE) of amphotericin B including a decrease in hemoglobin levels, electrolyte imbalance, renal toxicity, and hepatic toxicity. A detailed representation is given in Table 4.

**Table 4:** Adverse effects after administration of amphotericin in 62 patients AE, adverse event.

AE	AE reported in	Average time lag between amphotericin and appearance of AE	Mean value in those subjects	Resolution of abnormality
Low Hb	41 (66.13%)	15 days	-	41
Hypokalemia	52 (83.87%)	17.6 days	2.88 mmol/L	19
Hyponatremia	48 (77.42%)	12.8 days	130 mmol/L	26
Hyperuricemia	34 (54.84%)	14.5 days	65.2 mg/dL	23
High creatinine	38 (61.29%)	14.6 days	1.93 mg/dL	17

Surgical treatment

Surgical procedures were required in 53 (85.48%) patients. Functional endoscopic sinus surgery was the most common surgical procedure in 40 patients followed by maxillectomy in 13 and eye extraction in eight patients. Among all the patients, 11 (17.7%) patients underwent neurosurgical intervention, which includes frontal craniotomy in six patients and temporal craniotomy in five patients. Of the 53 patients who underwent surgical intervention, 35 patients were alive whereas 14 patients died (four subjects were lost to follow-up). A significant association (p=0.008) was discovered between surgical intervention and favorable prognosis.

Final outcome 

At the end of 12 weeks, only 18 (29.03%) subjects completely recovered without any residual signs and symptoms, while 19 (30.63%) patients had some persistent signs and symptoms. The most common residual symptoms were ptosis (six subjects), hemiplegia (five subjects), and facial symptoms like numbness/pain/swelling (four subjects). This indicates that a total of 37 (59.68%) subjects survived, while 21 (33.87%) patients died; four (6.45%) subjects were lost in the follow-up period. 

Factors responsible for the final outcome

To compare the factors that influence the outcome of cerebral mucormycosis, we divided our patients into two groups - survivors (n=37) and non-survivors (n=21). We were unable to trace four patients in the last week of our study and hence they are excluded. We did not find a strong impact of diabetes on the progression and outcome of the disease (this does not negate diabetes mellitus as a risk factor for mucormycosis). We found radiographic findings of cerebral infarction to have a strong association (0.0015) with the prognosis. In comparing patients being prescribed posaconazole vs those not being prescribed posaconazole, we found the step-down therapy to be a significant factor influencing the outcome (p-value=0.0005). Similarly, retrobulbar amphotericin (p-value=0.03) and surgical intervention (p-value=0.004) also depicted a significant association with the survival of the patients. Other factors are elaborated on in Table 5.

**Table 5:** Correlation of various factors associated with survival in patients suffering from cerebral mucormycosis (n=58) BMI, body mass index; ICA, internal carotid artery; ABLC, amphotericin B-lipid complex.

	Survivors (N=37)	Non-survivors (N=21)	p-Value (chi-square test)
Demographics			
Age	49.51±12.48 years	52.86±9.25 years	
Occupation			
Farmer/florist	12	8	0.67
Average BMI	24.78±4.04	22.87±3.46	
Smoking/tobacco	19	8	0.33
Diabetes mellitus			
Pre-COVID	16	9	0.98
Post-COVID (total)	31	18	0.85
COVID history	35	20	0.92
Hospitalization history	16	9	0.98
Drug treatment received	34	17	0.22
Steroids	24	13	0.82
Time lag between COVID and mucormycosis	18.69±9.59 days	17.63±7.41 days	
Neural symptom is seen in	20	12	0.82
Time lag between the onset of mucormycosis and the first neural symptom	22.75±18.15 days	19.46±14.13 days	
Radiological findings			
ICA thrombosis	9	4	0.64
Cavernous-sinus thrombosis	14	4	0.13
Meningitis	9	3	0.36
Acute infarcts	9	14	0.0015 (significant)
Surgical intervention	35	14	0.004 (significant)
Convention amphotericin	33	20	0.43
Liposomal amphotericin	22	9	0.22
ABLC	4	1	0.43
Retrobulbar amphotericin	7	0	0.03 (significant)
Posaconazole	34	11	0.0005 (significant)

## Discussion

The published data on mucormycosis rose exponentially after 2019, but till date, there are only selected studies that focus on only cerebral mucormycosis. We compared two such studies [7,16] and found our patient population to have a significantly higher survival rate, which we attribute to aggressive antifungal therapy and early surgical intervention. Administration of antifungal amphotericin B to all the patients and a step-down therapy of posaconazole was continued until negative biopsy results led to better elimination of the invasive fungal organism. Continuous monitoring of renal and liver function tests was also done to check for any nephrotoxicity due to amphotericin B, and subsequent efforts to control the adverse reactions included the administration of potassium chloride (KCl) and switching to liposomal amphotericin B. Thus, a combination of early detection and aggressive treatment along with proper time management of any possible side effects of the anti-fungal medications has led to a significant improvement in the outcome as compared to already existing literature. Other compared parameters are enlisted in Table 6.

**Table 6:** Comparison of demographics, risk factors, symptoms, anatomical locations, and pharmacotherapy of our study with studies by Ma J et al. [7] and Jiang N et al. [16] *These are retrospective studies, hence lack a specified follow-up period.

	Our study	Ma J [7]	Jiang N [16]	
Type of study	Mixed (retrospective and prospective) for 12 weeks	Retrospective*	Retrospective*	
No of cases	62	81	11	
Mean age	50.2 years	41.6 years	53.7 years	
Diabetes mellitus	83.87%	47%		-
Paranasal sinus involvement	96.78%	65.5%	72.73%	
Orbital invasion	70.97%	42%	100%	
Cerebral involvement				
Frontal	24.2%	39.5%	9.1%	
Temporal	50%	19.8%	-	
Cavernous sinus	30.6%	2.5%	-	
Meningitis	40.32%	29.6%	36.4%	
Treatment	Amphotericin (in 100% subjects) followed by posaconazole (in 30%)	Amphotericin 1 mg/kg followed by fluconazole	Amphotericin 0.3 mg/kg to 1 mg/kg gradually	
Surgery	84%	-	81.8%	
Survival rate	63.79%	23.5%; before 2000: 9.4%; after 2000: 50%	27.27%	

Despite having a large enough sample size for a rare disease like this, we believe there were several limitations too. Since cerebral mucormycosis is a potentially fatal disease, the total duration of the study could have been longer than 12 weeks. The sample size could have been larger. Conducting a multicentric study might have helped improve the overall statistical power and further reduced the changes of a beta error. Finally, being a cohort study, we did not have a control population (mucormycosis patients without cerebral extension), and such a study design can highlight risk factors for cerebral invasion of the fungal infection. However, to the best of our knowledge, this is the largest single-centric cohort study of exclusive cerebral mucormycosis patients till date.

## Conclusions

In this study, we emphasized that COVID-19 and diabetes mellitus were significant risk factors contributing to the development of mucormycosis. Common signs and symptoms of mucormycosis often appeared within a few weeks of COVID, but neurological manifestations in these patients were either absent or appeared relatively late. Hence, periodic radiological scans seemed the only reliable way to rule out the possibility of cerebral extension of mucormycosis. Even though cerebral mucormycosis had traditionally been one the most fatal conditions, we recorded a significantly high survival rate (about 60%) at the end of 12 weeks, attributing it to prompt surgical interventions and step-down posaconazole therapy following the amphotericin regimen. 

## References

[1] Chakrabarti A, Chatterjee SS, Das A (2009). Invasive zygomycosis in India: experience in a tertiary care hospital. Postgrad Med J.

[2] Raut A, Huy NT (2021). Rising incidence of mucormycosis in patients with COVID-19: another challenge for India amidst the second wave?. Lancet Respir Med.

[3] Avatef Fazeli M, Rezaei L, Javadirad E (2021). Increased incidence of rhino-orbital mucormycosis in an educational therapeutic hospital during the COVID-19 pandemic in western Iran: An observational study. Mycoses.

[4] Pradhan P, Shaikh Z, Mishra A (2021). Predisposing factors of rhino-orbital-cerebral mucormycosis in patients with COVID 19 infection. Indian J Otolaryngol Head Neck Surg.

[5] Petrikkos G, Skiada A, Lortholary O, Roilides E, Walsh TJ, Kontoyiannis DP (2012). Epidemiology and clinical manifestations of mucormycosis. Clin Infect Dis.

[6] Kumari A, Rao NP, Patnaik U (2021). Management outcomes of mucormycosis in COVID-19 patients: A preliminary report from a tertiary care hospital. Med J Armed Forces India.

[7] Ma J, Jia R, Li J (2015). Retrospective clinical study of eighty-one cases of intracranial mucormycosis. J Glob Infect Dis.

[8] Lowe JT Jr, Hudson WR (1975). Rhincerebral phycomycosis and internal carotid artery thrombosis. Arch Otolaryngol.

[9] Cornely OA, Alastruey-Izquierdo A, Arenz D (2019). Global guideline for the diagnosis and management of mucormycosis: an initiative of the European Confederation of Medical Mycology in cooperation with the Mycoses Study Group Education and Research Consortium. Lancet Infect Dis.

[10] Wali U, Balkhair A, Al-Mujaini A (2012). Cerebro-rhino orbital mucormycosis: An update. J Infect Public Health.

[11] Romero-Zamora JL, Bonifaz A, Sánchez CJ, Lagunas-Ramírez A, Hidalgo-Loperena H (2000). Rhinocerebral mucormycosis. Report of twelve cases. Rev Med Hosp Gen Mex.

[12] Schell WA (2000). Histopathology of fungal rhinosinusitis. Otolaryngol Clin North Am.

[13] Talmi YP, Goldschmied-Reouven A, Bakon M (2002). Rhino-orbital and rhino-orbito-cerebral mucormycosis. Otolaryngol Head Neck Surg.

[14] Government of India.Ministry Of Health and Family Welfare. Revised Guidelines on Clinical Management of COVID-19 (2022). Government of India. Ministry of Health and Family Welfare. Revised Guidelines on Clinical Management of COVID-19. http://www.mohfw.gov.in/pdf/RevisedNationalClinicalManagementGuidelineforCOVID1931032020.pdf.

[15] Francone M, Iafrate F, Masci GM (2020). Chest CT score in COVID-19 patients: correlation with disease severity and short-term prognosis. Eur Radiol.

[16] Jiang N, Zhao G, Yang S (2016). A retrospective analysis of eleven cases of invasive rhino-orbito-cerebral mucormycosis presented with orbital apex syndrome initially. BMC Ophthalmol.

